# A predicted transmembrane region in plant diacylglycerol acyltransferase 2 regulates specificity toward very-long-chain acyl-CoAs

**DOI:** 10.1074/jbc.RA120.013755

**Published:** 2020-09-01

**Authors:** Simon Jeppson, Helena Mattisson, Kamil Demski, Ida Lager

**Affiliations:** 1 Department of Plant Breeding, Swedish University of Agricultural Sciences, Alnarp, Sweden; 2 Intercollegiate Faculty of Biotechnology, University of Gdańsk and Medical University of Gdańsk, Gdańsk, Poland

**Keywords:** plant lipids, Brassica napus, acyltransferase, enzyme specificity, Kennedy pathway, DGAT2, DAG, TAG, lipid, diacylglycerol, triacylglycerol, plant biochemistry

## Abstract

Triacylglycerols are the main constituent of seed oil. The specific fatty acid composition of this oil is strongly impacted by the substrate specificities of acyltransferases involved in lipid synthesis, such as the integral membrane enzyme diacylglycerol acyltransferase (DGAT). Two forms of DGAT, DGAT1 and DGAT2, are thought to contribute to the formation of seed oil, and previous characterizations of various DGAT2 enzymes indicate that these often are associated with the incorporation of unusual fatty acids. However, the basis of DGAT2's acyl-donor specificity is not known because of the inherent challenges of predicting structural features of integral membrane enzymes. The recent characterization of DGAT2 enzymes from *Brassica napus* reveals that DGAT2 enzymes with similar amino acid sequences exhibit starkly contrasting acyl-donor specificities. Here we have designed and biochemically tested a range of chimeric enzymes, substituting parts of these *B. napus* DGAT2 enzymes with each other, allowing us to pinpoint a region that dramatically affects the specificity toward 22:1-CoA. It may thus be possible to redesign the acyl-donor specificity of DGAT2 enzymes, potentially altering the fatty acid composition of seed oil. Further, the characterization of a DGAT2 chimera between *Arabidopsis* and *B. napus* demonstrates that the specificity regulated by this region is transferrable across species. The identified region contains two predicted transmembrane helices that appear to reoccur in a wide range of plant DGAT2 orthologues, suggesting that it is a general feature of plant DGAT2 enzymes.

Oilseeds produce large quantities of triacylglycerol (TAG), which accumulates in the seed oil during seed development. The seed oil constitutes a highly condensed carbon and energy storage dedicated to the emerging seedling during seed germination (1). Plants assemble TAG in the endoplasmic reticulum from glycerol-3-phosphate and fatty acids (FAs) that originates from the plastids (2). The Kennedy pathway outlines the assembly of a wide range of lipids, including the acyl-CoA–dependent formation of TAG. Acyltransferases in the Kennedy pathway utilize acyl-CoA substrates and are responsible for the sequential acylation of a glycerol backbone (3). Diacylglycerol acyltransferase (DGAT) is the sole enzyme committed to TAG assembly in the Kennedy pathway and catalyzes the formation of TAG from diacylglycerol (DAG) and acyl-CoA. An additional acyltransferase, phospholipid:diacylglycerol acyltransferase, is involved in seed oil accumulation. Phospholipid:diacylglycerol acyltransferase catalyzes the acyl-CoA–independent formation of TAG and utilizes an acyl moiety from PC to acylate DAG into TAG (4).

Plants can produce several hundred different FAs. Many of these FAs are highly suited for a wide range of industrial applications. However, many conventional seed oils only contain a few reoccurring common FAs (5).

Three distinct isoforms of DGAT are present in plants, and they differ starkly from each other in many regards despite their shared catalytic capability. DGAT1 and DGAT2 are both integral enzymes embedded in the membranes of the endoplasmic reticulum (6). Enzymes of the DGAT1 isoform are generally constituted by longer polypeptide chains than DGAT2 (7). Further, predictions usually indicate that DGAT1 polypeptide sequences contain more transmembrane helices than DGAT2, between 6 and 9 compared with 1 or 2 (8). There is no resemblance between the DNA or amino acid sequences of DGAT1 and DGAT2 isoforms, and they are thus probably the result of convergent evolution (8). Both isoforms are capable of channeling nascent fatty acids to the accumulating TAG in developing seeds (9–11). The third isoform of DGAT, DGAT3, is soluble and is yet to be confirmed to be involved in seed oil accumulation (12, 13).

DGAT2, first identified in an oleaginous fungus, *Mortierella ramanniana* (Möller) Linnem, has since been identified in a wide range of plant species (14). Several plant DGAT2 enzymes are capable of incorporating unusual FAs into TAG as has been demonstrated with DGAT2 enzymes from, for example, castor bean (*Ricinus communis* L.) and the tung tree (*Vernicia fordii* (Hemsl.) Airy Shaw) (6, 11). Castor bean produces a seed oil rich in hydroxy FAs (HFAs). *Arabidopsis* seed oil is devoid of HFA, but the concurrent expression of castor bean DGAT2 and a castor bean fatty acid hydroxylase in transgenic *Arabidopsis*, where the hydroxylase modifies FAs into HFAs and the DGAT2 enzyme incorporates the HFAs into TAG, results in ample amounts of HFA in the seed oil, up to 30 mol%. Transgenic *Arabidopsis* expressing the hydroxylase alone produces a seed oil containing approximately half of that amount of HFA, 17 mol%, which demonstrate the significant impact DGAT2 enzymes may have on seed oil FA composition (15). The substrate specificity of DGAT2 enzymes is therefore of great importance because it may significantly affect the TAG FA composition. The apparent capability of these DGAT2 to incorporate unusual FAs into TAG is of high interest because many unusual FAs are suitable for a wide range of industrial applications.

The folding structure of DGAT2 is, to a large extent, yet unknown because of the huge challenges presented by purification, crystallization, and the prediction of integral proteins structure (16). Modifications of DGAT enzymes have so far mainly focused on improving the catalytic activity and substrate specificities of DGAT1 (17–20). To our knowledge, no prior studies have focused on sequence motifs affecting the substrate specificity in DGAT2.

Functional studies of the *Saccharomyces cerevisiae* DGAT2 orthologue, Dga1p, reveal the importance of two motifs: YFP and HPHG (depicted by their amino acid presence). Mutations in either of the two motifs abolish the DGAT enzymatic activity. EPHS substitutes the HPHG motif in plant-derived DGAT2 orthologues. Many plant-derived DGAT2 orthologues are functional when expressed in recombinant yeast despite harboring the EPHS motif, yet a substitution of HPHG with EPHS in the yeast Dga1p renders an inactive enzyme (21).

Concepts to produce novel designed oil qualities in oil crops have often resulted in plants with impaired oil content or suboptimal levels of the desired FAs in the seed oil. Some of these discouraging attempts may be, at least in part, caused by incompatibility between the desired FAs and the substrate specificity of acyltransferases or other enzymes involved in lipid metabolism. Crambe (*Crambe hispanica* subsp. *abyssinica* (Hochst. ex R.E.Fr.) Prina) seed oil contains ∼60% of erucic acid, but an even higher amount is desirable (22). An ambitious strategy with three distinct approaches, all aiming at increasing the erucic acid content in the seed oil, resulted in less than optimal results (23). The strategy implemented to increase the erucic acid content in crambe includes the introduction of a lysophosphatidic acid acyltransferase from *Limnanthes douglasii* R. Br., capable of using 22:1-CoA as substrate (22). The strategy also includes the introduction of a *Brassica napus* L. elongase capable of producing erucic acid from 18:1 and an RNAi targeting a Δ^12^ desaturase that competes with the elongase for the 18:1 precursor (22). Transgenic crambe achieves a significant increase of di-22:1-DAG with these three components working in concert, yet additional bottlenecks seem to hamper significant increases in trierucin (24). Studies of the crambe DGAT specificities indicate that these, especially the DGAT2 forms, are unsuitable for the synthesis of trierucin because of their substrate specificity (25). The substrate specificity of acyltransferases involved in the synthesis of lipids is therefore critical and must be taken into consideration while attempting to introduce a novel FA composition in seed oil.

A recent study of *B. napus* DGAT includes *in vitro* assays of *Bna*.DGAT2 using a broad range of acyl-CoA and di-caproyl-DAG (di-6:0-DAG) as substrates (26). All four investigated *Bna*.DGAT2 forms exhibit high specificity toward 18:3-CoA, and two of them also have a high specificity toward 22:1-CoA. The two forms that are highly specific toward 18:3-CoA, henceforth denoted as 18:3-CoA–specific, have highly similar amino acid sequences, as have the two other forms, specific to both 18:3-CoA and 22:1-CoA, henceforth denoted as 22:1-CoA–accepting. The two groups share 98% amino acid sequence identity within their respective group and 78–80% between the two groups (26). *B. napus* is amphidiploid and contains two genomes, genome A (2n = 20) from *Brassica rapa* L. and C (2n = 18) from *Brassica oleracea* L. (27). The *DGAT2* genes of *B. napus* are thus homoeologues (28, 29). The *B. napus* genome was recently reannotated, and the DGAT2 genes are now reported to reside on four individual chromosomes A1, A3, C1, and C7 according to NCBI, whereas the previous annotation positioned all of them in A chromosomes. Each ancestral genome, A and C, harbor one DGAT2 of each specificity type: 18:3-CoA–specific and 22:1-CoA–accepting. Despite the recent reannotation, the genes and enzymes maintain the nomenclature from the previous publication to ensure consistency. The *Bna*.DGAT2 genes and corresponding enzymes are named according to Ref. 30. The gene nomenclature within *Brassica* species is standardized. Genes derived from *B. napus* start with *Bna*, followed by a capital letter indicating their genomic ancestry: A, C, or X (for unknown). The terminology also includes the gene name and locus denomination. Hence, the gene *Bna*A.DGAT2.b is the full name for a DGAT2 gene from *B. napus* that resides on a chromosome from the ancestral A genome at locus b.

The two groups of DGAT2 in *B. napus* with similar amino acid sequences and highly distinct substrate specificities provide an excellent opportunity for studies of DGAT2 acyl-CoA substrate specificity motifs. Here, a yeast strain incapable of producing TAG, H1246, is used to determine the substrate specificity of the *Bna*.DGAT2 using microsomal preparations of recombinant H1246 expressing the *Bna*.DGAT2 orthologues. We investigate the nature of acyl-donor specificity in *Bna*.DGAT2 through chimeric enzymes based on an enzyme of the 18:3-CoA–specific type and a member of the 22:1-CoA–accepting type. Swapping of two predicted closely situated transmembrane regions results in a changed specificity, which strongly indicates that this region is of great importance for acyl-donor specificity. Alignment and prediction of transmembrane helix occurrence in a multitude of plant DGAT2 sequences reveal a consensus in which two closely situated transmembrane helices reside close to the N terminus. Substitution of the identified region in *Arabidopsis* DGAT2 with the corresponding region from a 22:1-CoA–accepting *B. napus* DGAT2 results in altered substrate specificity. This change indicates that the importance of this region for substrate specificity is not merely confined to *B. napus* DGAT2 enzymes.

## Results

**Figure 1.**
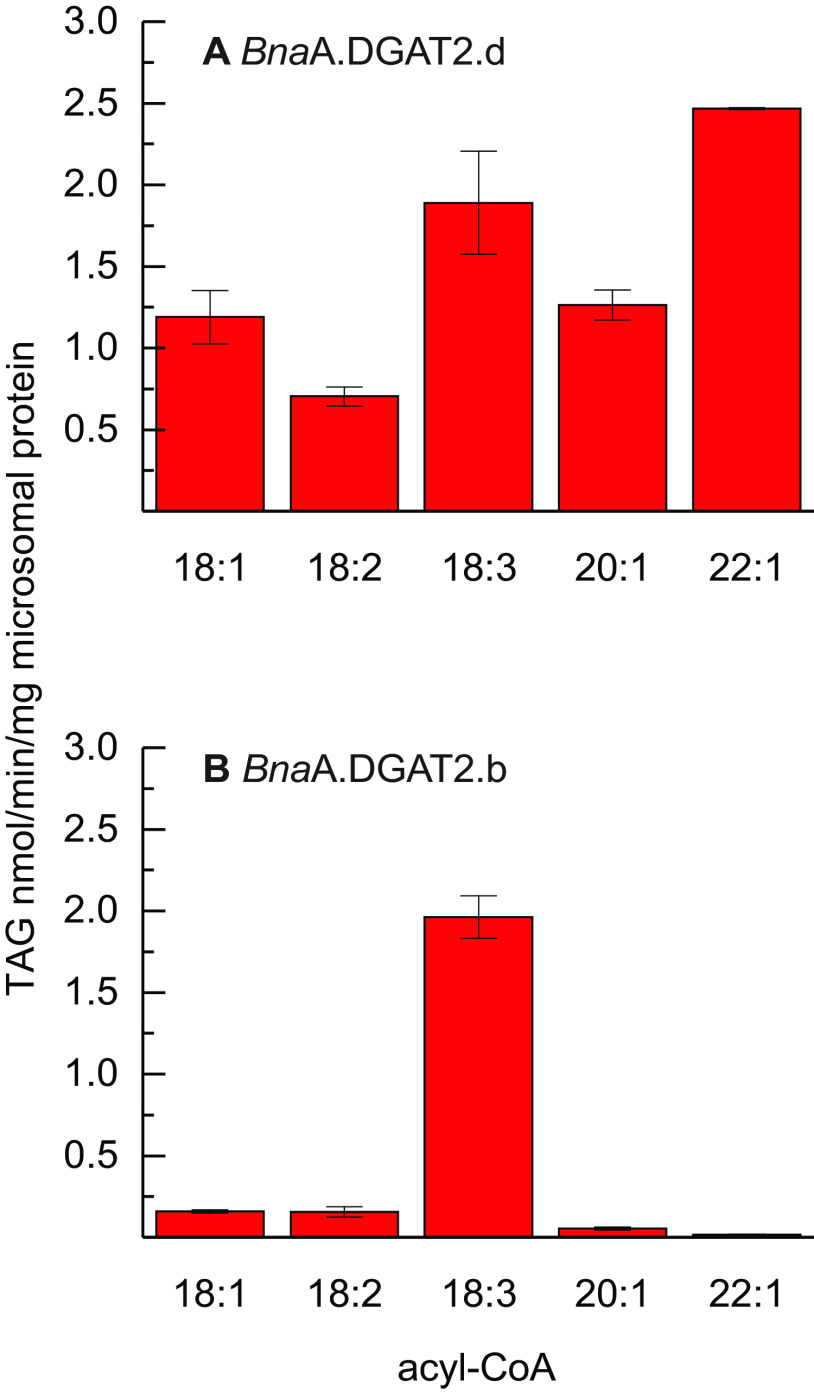
**Acyl-donor specificity of recombinant yeast microsomal preparations when assessed with di-6:0-DAG.** *A*, *Bna*A.DGAT2.d. *B*, *Bna*A.DGAT2.b. The *error bars* denote standard deviation (*n* = 3).

We determined the acyl-donor specificity of *Bna*A.DGAT2.b and *Bna*A.DGAT2.d in microsomal preparations of recombinant yeast using [^14^C]di-6:0-DAG as an acyl-acceptor. Further, we assessed the specificities of all enzymes, both the parental wildtypes and the chimeras under the same conditions, 40 μg of microsomal protein during 30 min of incubation. We chose to prioritize the same assay conditions for all enzymes instead of measuring under linear conditions. The WT enzymes differ starkly in their specificity toward 22:1-CoA relative to 18:3-CoA, although they originate from the same plant species. Both enzymes synthesize similar amounts of TAG when provided with 18:3-CoA. *Bna*A.DGAT2.b exhibits a high specificity toward 18:3-CoA compared with all other acyl donors investigated. *Bna*A.DGAT2.d, on the other hand, produces TAG at similar levels when provided 22:1-CoA as in assays supplemented with 18:3-CoA (Fig. 1). *Bna*A.DGAT2.d also exhibits a broader acceptance of other tested acyl donors.

**Figure 2.**
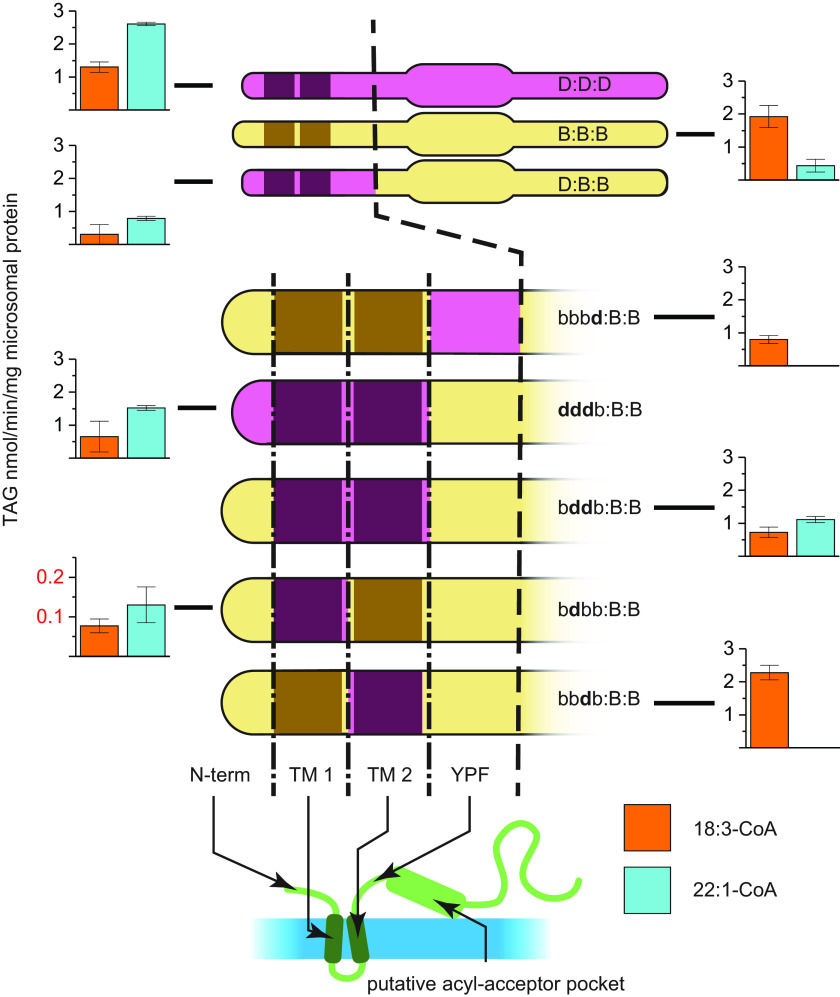
**Schematic overview of *Bna*A.DGAT2.d, *Bna*A.DGAT2.b, and chimeric enzymes and their respective specificity toward 18:3-CoA and 22:1-CoA.** *Purple*, *Bna*A.DGAT2.d; *yellow*, *Bna*A.DGAT2.b. Enzyme illustrations and features are not true to scale. *Colons* delimit the amino acid into thirds of the amino acid sequence, and a *capital letter B* or *D* refers to either *Bna*A.DGAT2.b or *Bna*A.DGAT2.d, respectively. *Lowercase letter b* or ***d*** represents subsegments of that third (where ***d*** is *bold* to more easily distinguish it from *b*). The enlarged section of the enzymes indicates a putative acyl-acceptor pocket motif. The segment containing the N terminus (*N-term*) is divided into four subsegments, indicated with *vertical dot–dash lines*. These subsegments contain the N terminus, two predicted transmembrane helices, and an amino acid sequence, YFP. The YFP motif is essential for maintaining the catalytic activity in the yeast DGAT2 orthologue Dga1p (21). *Error bars* denote standard deviation (*n* = 3).

We designed a range of chimeric enzymes to elucidate whether a particular region in the DGAT2 amino acid sequence affects acyl-donor specificity. These chimeric enzymes have a backbone from the 18:3-CoA–specific *Bna*A.DGAT2.b where large segments from the 22:1-CoA–accepting *Bna*A.DGAT2.d substitute the corresponding amino acid sequences. We assessed changes in the specificity of the chimeric enzyme compared with *Bna*A.DGAT2.b in biochemical assays containing microsomal preparations supplemented with either 18:3-CoA or 22:1-CoA and [^14^C]di-6:0-DAG. An increased specificity toward 22:1-CoA relative to 18:3-CoA indicates an influence on specificity by the *Bna*A.DGAT2.d sequence substitution. We implemented a naming convention for the chimeric enzymes based on the following rules; a colon delimits the amino acid into thirds of the entire amino acid sequence where a capital letter B or D refers to either *Bna*A.DGAT2.b or *Bna*A.DGAT2.d, respectively. Lowercase letters b or **d** represents subsegments of that third (where **d** is bold to more easily distinguish it from b). Thus, D:B:B, representing a chimera in which *Bna*A.DGAT2.d substitutes the first third of *Bna*A.DGAT2.b and **d**bbb:B:B, is only substituted in the first part of the first third. The screening of these chimeric enzymes specificity reveals that the specificity signature of *Bna*A.DGAT2.d coincides with the first third of the enzyme (Fig. 2, *top three illustrations*).

Next step was to focus on the first third of the enzymes, which we divided into four regions: N terminus, first and second predicted transmembrane helices, and a stretch containing the YPF motif (see Fig. 2, *bottom five enzymes*). Only a few amino acids separate the predicted transmembrane helices in the parental WT enzymes. Iterations of the chimeric enzyme substituting either of the regions in the first third or combinations thereof in the *Bna*A.DGAT2.b provide further information about acyl-donor specificity. Different combinations of chimeric enzymes were constructed and expressed in yeast for characterization of their relative activity toward 18:3-CoA and 22:1-CoA (Fig. 2). Substitution of the first segment containing the N terminus in *Bna*A.DGAT2.b results in a chimeric enzyme that produces no detectable levels of TAG with either acyl-CoA (data not shown). It is thus difficult to draw any conclusion of the impact of this region on acyl-donor specificity. However, chimeric enzymes without substituting the N terminus of the *Bna*A.DGAT2.b exhibit a significantly altered acyl-CoA specificity (Fig. 2). All the iterated chimeric enzyme designs that contain the first transmembrane helix from *Bna*A.DGAT2.d also maintain a relatively high preference for 22:1-CoA compared with 18:3-CoA, indicating that this region is of importance for acyl-CoA specificity (Fig. 2). The chimeric enzyme b**d**bb:B:B, substituted only at the first transmembrane helix, has, however, a drastically reduced catalytic activity. Substitution of the second transmembrane helix alone (bb**d**b:B:B; Fig. 2) retains DGAT catalytic activity but does not affect the acyl acceptor specificity. The chimeric enzyme b**dd**b:B:B, in which both transmembrane helices are substituted, maintains the ability to form TAG but with an altered specificity that more closely resembles that of *Bna*A.DGAT2.d instead of *Bna*A.DGAT2.b (Fig. 2).

**Figure 3.**
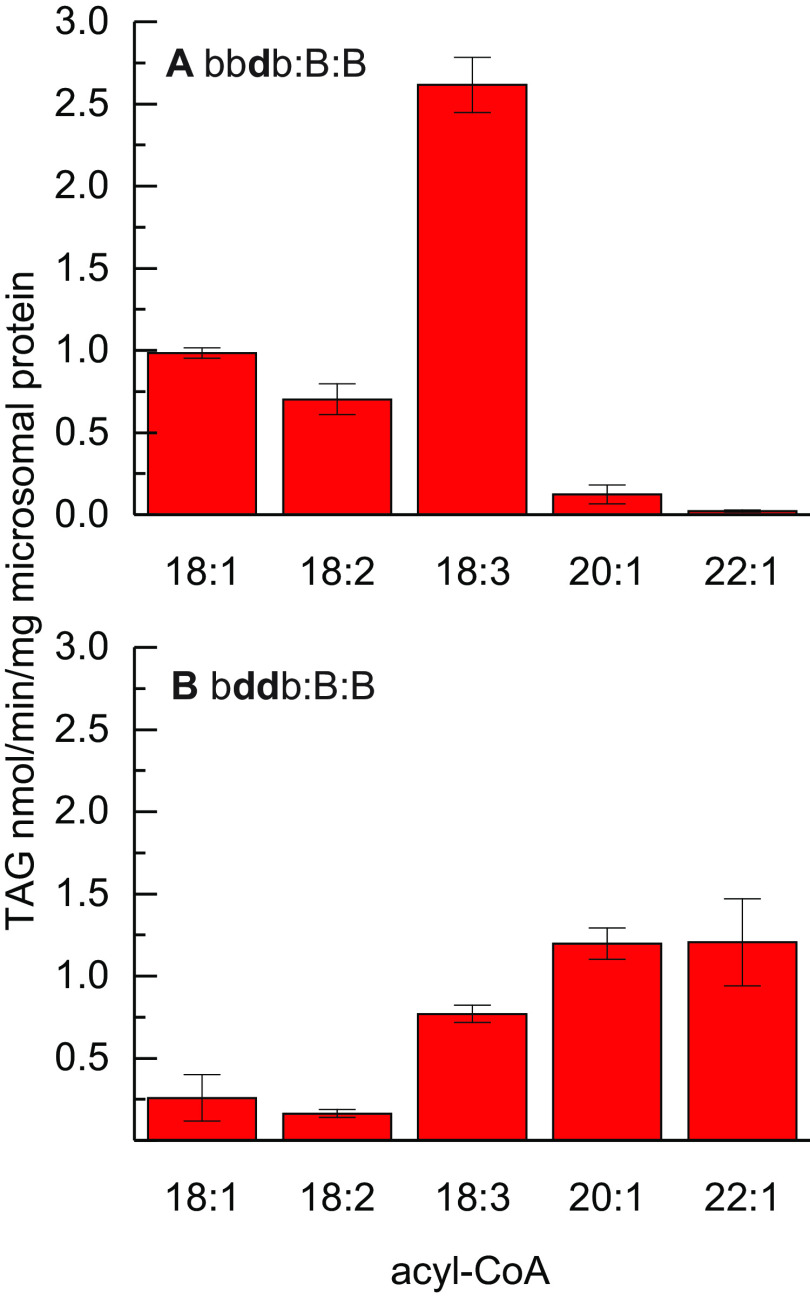
**Acyl-donor specificity of recombinant yeast microsomal preparations when assessed with [^14^C]di-6:0-DAG.** *A*, bb**d**b:B:B. *B*, b**dd**b:B:B. *Error bars* denote standard deviation (*n* = 3).

We further characterized the acyl-CoA specificity of two chimeric enzymes, bb**d**b:B:B and b**dd**b:B:B, with 18:1-, 18:2-, 18:3-, 20:1-, and 22:1-CoA (Fig. 3). Both chimeric enzymes contain fragments of *Bna*A.DGAT2.d in *Bna*A.DGAT2.b, which substitutes either the second transmembrane helix or both, and maintain a catalytic activity with 18:3-CoA comparable with the WT parental enzymes. However, the acyl-CoA specificity of bb**d**b:B:B, previously perceived as unaltered during the screening process, differs from *Bna*A.DGAT2.b with 18:1-CoA and 18:2-CoA substrates, which are more similar to the *Bna*A.DGAT2.d (Figs. 1 and 3*A*), but, it does not gain any increased specificity toward either 20:1-CoA or 22:1-CoA. The second enzyme characterized in detail, b**dd**b:B:B, demonstrates a clear preference for very-long-chain acyl-CoAs, and the specificity appears to approximately resemble that of *Bna*A.DGAT2.d (Figs. 1 and 3*B*).

**Figure 4.**
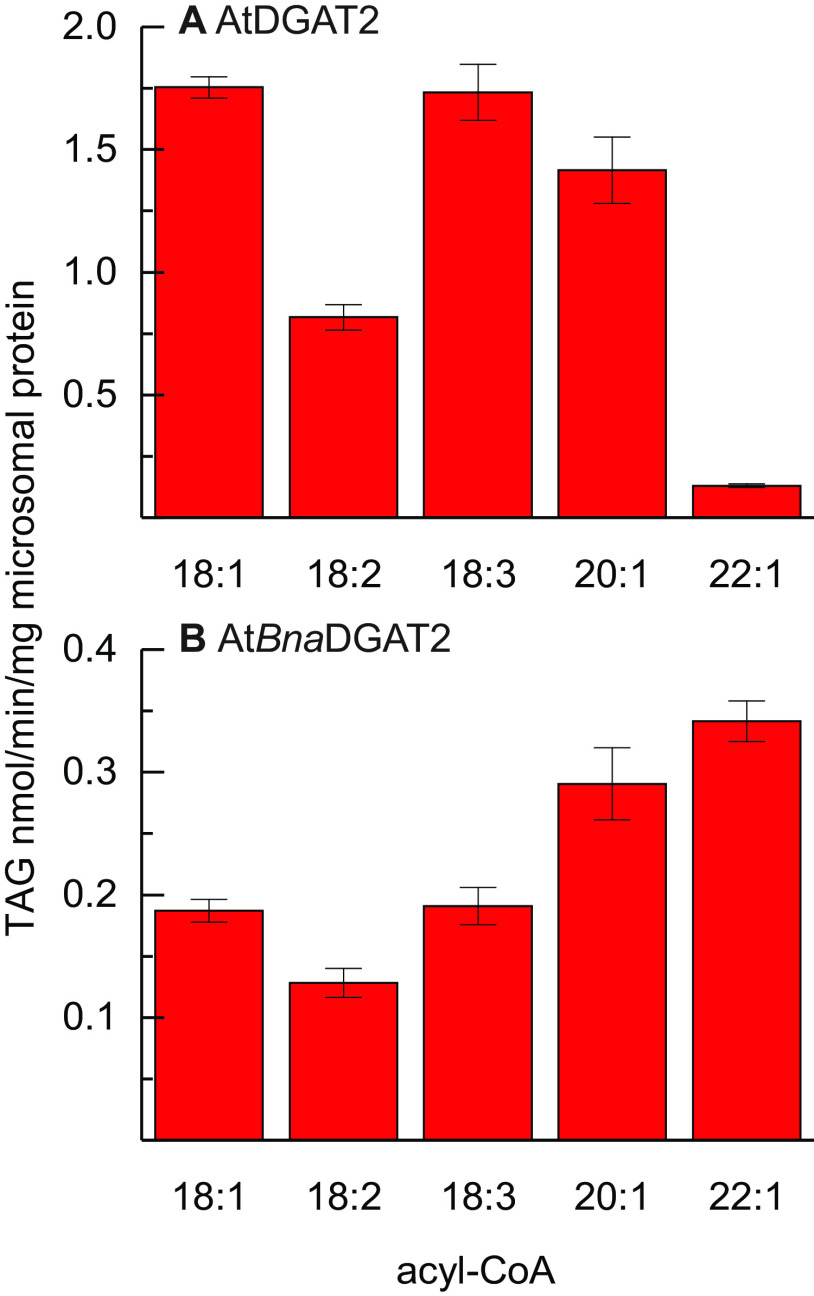
**Acyl-donor specificity of recombinant yeast microsomal preparations when assessed with [^14^C]di-6:0-DAG.** *A*, WT parental enzyme AtDGAT2. *B*, chimera At*Bna*DGAT2. *Error bars* denote standard deviation (*n* = 3).

We assessed acyl-donor specificity of a chimera between AtDGAT2 and *Bna*A.DGAT2.d to conclude whether the identified regions impact on acyl-donor specificity confines to DGAT2 enzymes from *B. napus* or whether it is a universal feature of plant DGAT2 enzymes. AtDGAT2 constitutes the backbone of the chimera, substituted with a segment from the 22:1-CoA–accepting enzyme, *Bna*A.DGAT2.d restricted to exclusively contain the two predicted transmembrane helices, and the few amino acids in between. The acyl donor substrate specificity of the chimeric At*Bna*.DGAT2 enzyme differs starkly from that of the parental WT AtDGAT2 (Fig. 4). The chimera expresses a high relative activity with 22:1-CoA, whereas the native AtDGAT2 utilizes this substrate poorly. However, the chimera's catalytic activity is significantly lower when compared with the WT (Fig. 4).

**Figure 5.**
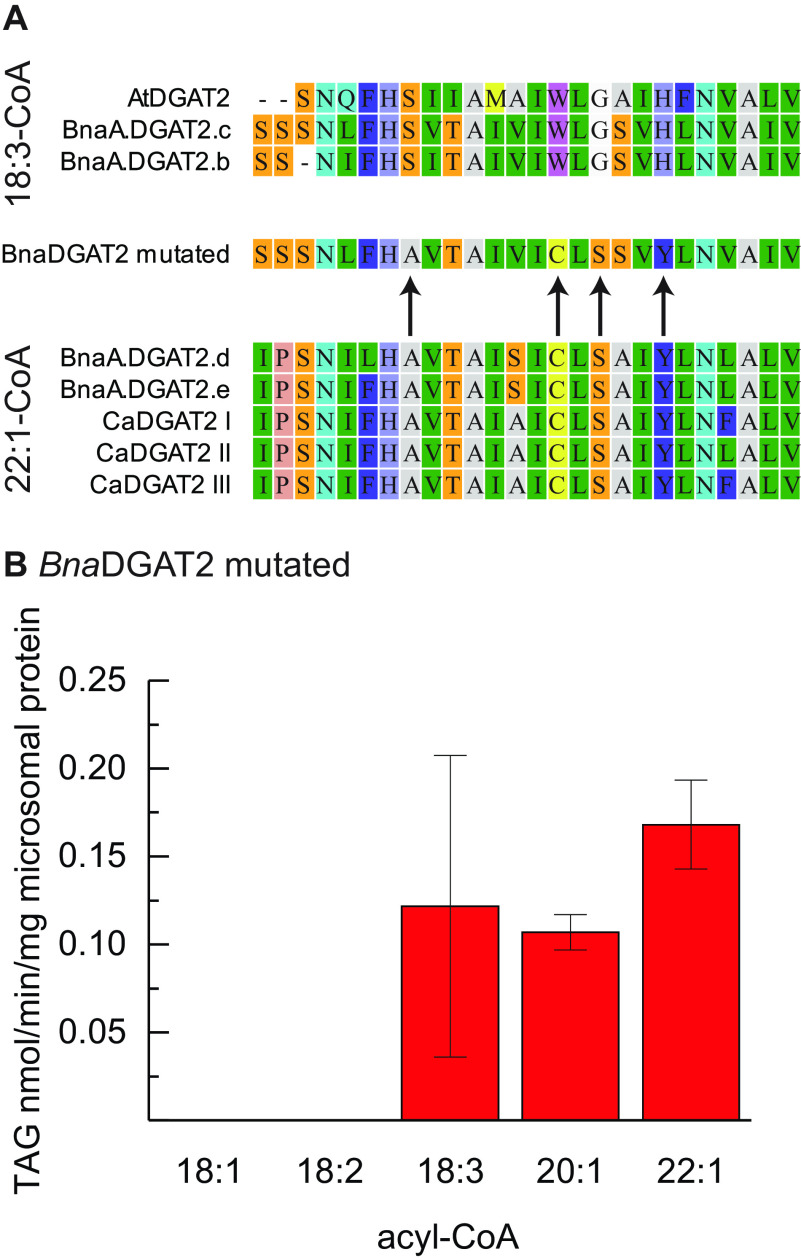
*A*, alignment of the predicted first transmembrane helix from eight DGAT2 amino acid sequences grouped by their type of acyl-donor specificity type, either 18:3 specific or 22:1 accepting, including *Bna*DGAT2-mutated sequence in between. *Arrows* indicate consensus at the amino acid level within a group, which is in contrast with consensus in the other group of DGAT2. *Bna*DGAT2-mutated corresponds to BnaA.DGAT2.b, where these amino acids are substituted. *B*, acyl-donor specificity of *Bna*DGAT2-mutated. *Error bars* denote standard deviation (*n* = 3).

We aligned a range of amino acid sequences with Clustal Omega including similar DGAT2 enzymes with contrasting specificity toward 22:1-CoA, which includes sequences of crambe DGAT2s, CaDGAT2 I-III, AtDGAT2, and *Bna*A.DGAT.b-e (25, 26). Grouping the enzymes according to their specificities, 18:3-CoA–specific or 22:1-CoA–accepting, reveals that four amino acids within the first predicted transmembrane helix are in consensus within the group but in contrast with the consensus in the other group (Fig. 5*A*). We thus designed an additional chimera, *Bna*DGAT2-mutated, similar to the *Bna*A.DGAT2.b enzyme but in which these four amino acids are substituted. The acyl-donor specificity assessment of *Bna*DGAT2-mutated indicates that the specificity is strongly affected compared with *Bna*A.DGAT2.b (Fig. 1*B* and 5*B*). The *Bna*DGAT2-mutated enzyme completely loses the ability to utilize 18:1-CoA and 18:2-CoA as acyl donors. Further, the *Bna*DGAT2-mutated specificity toward 22:1-CoA increases drastically relative to 18:3-CoA when compared with *Bna*A.DGAT2.b. However, the catalytic activity is drastically reduced, just as in the b**d**bb:B:B chimera (Fig. 2).

**Figure 6.**
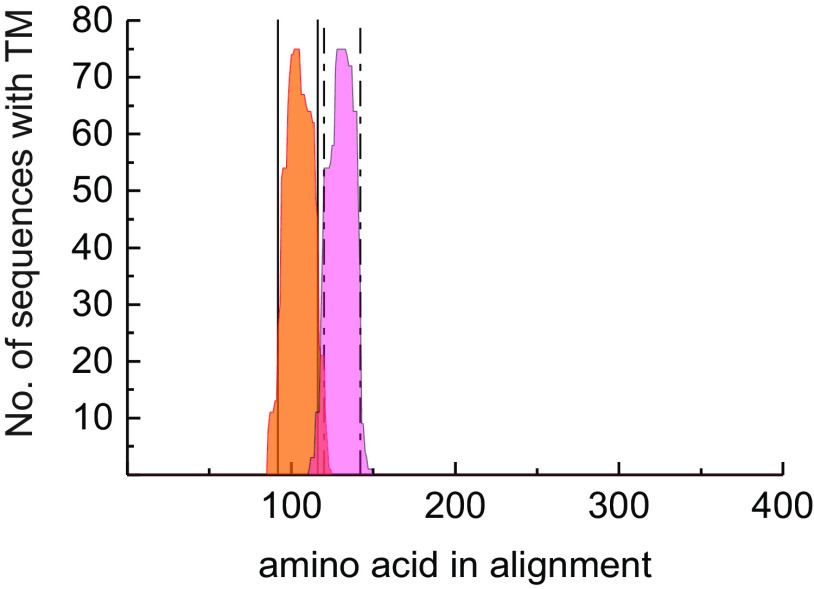
**Frequency of predicted transmembrane helices as predicted by TMHMM aligned by Clustal Omega in 77 DGAT2 orthologues from plants.** The *orange histogram* corresponds to the first transmembrane helix, and the *pink histogram* represents the second transmembrane helix.

The WT *Bna*.DGAT2 enzymes in this study are highly similar and share several features. TMHMM predicts that both enzymes harbor two transmembrane helices in the vicinity of the N terminus (31). The chimeric enzyme designs reveal that the acyl-CoA specificity largely coincides with the two predicted transmembrane helices. A previous study of DGAT enzymes includes a transmembrane prediction of five plant DGAT2s (8). These enzymes seem to contain two transmembrane regions at approximately the same place as the *Bna.*DGAT2 of this study, as are DGAT2 forms previously identified in crambe (25). A wide range of putative plant DGAT2 sequences has become publically available since the identification of the first DGAT2. We performed alignment and prediction of transmembrane helices of several DGAT2 to conclude whether this feature is generally abundant in plant-derived DGAT2. Prediction of transmembrane helices of 77 amino acids sequences from plants similar to *Arabidopsis* DGAT2 (NP_566952.1) by TMHMM and subsequent alignment with Clustal Omega indicate that 75 of 77 indeed may contain two closely situated transmembrane helices in the same predicted region as the *Bna.*DGAT2 enzymes (Fig. 6 and Fig. S1).

We have here, through several chimeric DGAT2 enzymes with WT parents within *B. napus*, demonstrated that acyl-donor specificity largely coincides with two putative transmembrane helices situated in the first third of the amino acid sequence, which commonly occurs in plant orthologues of DGAT2. It does not seem to be an isolated feature restricted to merely the *B. napus* DGAT2 enzymes, because the specificity of a chimera between *B. napus* and *Arabidopsis* changes similarly.

## Discussion

The acyl-donor specificity of parental WT enzymes *Bna*A.DGAT2.b and *Bna*A.DGAT2.d determined here is similar to the specificity previously published by (26). *Bna*A.DGAT2.b is highly specific toward 18:3-CoA but not toward 22:1-CoA, whereas *Bna*A.DGAT2.d utilizes these substrates at similar levels. Subtle differences in acyl specificity between our findings and that previously reported do occur, as expected because we use different protein concentrations, incubation times, and microsomal isolation events.

The assessed specificity of the chimeric enzymes designed from the two enzymes strongly indicates that the acyl-donor specificity largely coincides with the two predicted transmembrane helices of *Bna*A.DGAT2.b and 2.d. Substitution of either of the two transmembrane helices affects the specificity of the chimeric enzymes. The first transmembrane helix of *Bna*A.DGAT2.d seems to provide means for incorporation of 20:1-CoA and 22:1-CoA in the chimeric enzymes. The second transmembrane helix of *Bna*A.DGAT2.d also affects the acyl-donor specificity in these chimeric enzymes, but in a different way than the first. Substitution of the second transmembrane helix alone in *Bna*A.DGAT2.b results in a noticeable increase of *de novo* formed TAG in assays provided with 18:1-CoA or 18:2-CoA compared with 18:3-CoA. Substitution of both transmembrane helices is potentially synergistic, and the combination provides a substrate specificity that allows efficient incorporation of 20:1-CoA and 22:1-CoA into TAG.

A topological and structural study of the murine DGAT2 enzyme reveals that this enzyme, just like the enzymes included here, contains two neighboring transmembrane helices situated in the first third of the protein (32). The bilayer membrane embeds the few amino acids between the transmembrane helices, and interestingly, the first transmembrane helix contains a domain, a putative neutral lipid-binding motif (32). The motif described in Ref. 32, FL*X*L*XXX^n^*, where *n* is any neutral amino acid, is not present in the enzymes investigated. The reverse of the putative neutral lipid-binding motif, *^n^XXX*L*X*LF, however, is present in the transmembrane region of both AtDGAT2 and *Bna*A.DGAT2.d. Both parental WT *B. napus* DGAT2 enzymes and AtDGAT2 contain the previously mentioned YPF motif and the EPHS motif, which frequently exist in plant DGAT2 orthologues. These enzymes also contain a conserved domain, a putative acyl-acceptor pocket motif, approximately in the middle of the sequence (33, 34).

The acyl-donor specificity profile of the chimeric b**dd**b:B:B enzyme, in which the two transmembrane regions of *Bna*A.DGAT2.d replace the corresponding regions from *Bna*A.DGAT2.b, does not perfectly reproduce the specificity profile of *Bna*A.DGAT2.d, yet there is a good resemblance between the two. At least much more so than between b**dd**b:B:B and *Bna*A.DGAT2.b. Both of the chimeric enzymes, b**dd**b:BB and At*Bna*DGAT2, harbor the two predicted transmembrane helices from *Bna*A.DGAT2.d, and these exhibit highly similar acyl-donor specificity patterns, but at different absolute levels. The identified region clearly affects the specificity toward 22:1-CoA in proportion to 18:3-CoA in a predictable manner when assessed with di-6:0-DAG in these chimeric enzymes. However, a redefinition of the identified region may be necessary, to perfectly replicate the acyl-donor specificity of a WT donor enzyme.

In this context, it should be mentioned that we use di-6:0-DAG, an artificial acyl acceptor, in our assays. Accumulating evidence indicates that determination of DGAT acyl-donor specificity with the artificial di-6:0-DAG does not necessarily reflect the specificity when assessed with long-chain acyl DAG species (25). Further investigation is needed to elucidate whether the differences in acyl-CoA specificities that we see between enzymes and different chimeras here also reflects the acyl-CoA specificities with long-chain DAG as acyl acceptor.

Complete substitution of the whole region as described here or even of a redefined region may not be necessary to alter the acyl-donor specificity of a DGAT2 enzyme. An insertion mutation in the genome of maize, *Zea mays* L., illustrates that single amino acids may affect both the FA composition and oil content in seeds. A single insertion of phenylalanine in maize DGAT1-2 at position 469 drastically increases the embryonic TAG accumulation. Maize lines that carry the alternative mutated DGAT, DGAT1-2 + Phe^469^, also display an altered FA composition of TAG compared with those that harbor the conventional DGAT1-2, in which oleic acid increases and linoleic acid decreases (20).

The alignment of the first predicted transmembrane helix, including five 22:1 accepting enzymes and three 18:3 specific enzymes, revealed four promising substitutions. The first substitution in *Bna.*DGAT2-mutated is Ser to Ala, this substitution changes from a neutral, polar amino acid into a neutral, nonpolar amino acid. The two following substitutions, Trp to Cys and Gly to Ser, change in the opposite direction from neutral, nonpolar amino acids into neutral, polar amino acids. The last substitution, His to Tyr, changes from a basic, polar amino acid into a neutral, polar amino acid. Hence, the selected amino acid substitutions also introduce significant changes in the properties of the selected amino acids. The contrasting properties introduced by these few substitutions in *Bna*A.DGAT2.b (*Bna*DGAT2-mutated) is sufficient to achieve significant changes in acyl-donor specificity compared with the native enzyme.

Both b**d**bb:B:B and *Bna*DGAT2-mutated have reduced catalytic activity in our assays when compared with the other investigated enzymes. The underlying reason for the reduced catalytic DGAT activity in these microsomal membranes is still unresolved. However, the majority of the other chimeric enzymes, for example, b**dd**b:B:B, produce TAG at similar levels when compared with the native enzymes. There may thus be essential features that enhance the catalytic capability of 22:1 accepting enzymes still unidentified within the predicted second transmembrane region. Unfortunately, there are no additional distinct differences between the two types of enzymes within the two predicted transmembrane helices. It may therefore be significantly more challenging to identify which amino acids restore the catalytic activity to comparable levels of the WT parental enzymes.

The chimeric At*Bna*DGAT2 enzyme, substituted in the two predicted transmembrane regions with that from *Bna*A.DGAT2d exhibits a drastic increase in the relative specificity toward 22:1-CoA compared with the WT *Arabidopsis* DGAT2 enzyme. This change in specificity demonstrates that the acyl-CoA specificity can be changed predictably between enzymes from different species. However, At*Bna*DGAT2 has a similar reduction in TAG production as b**d**bb:B:B and *Bna*DGAT2-mutated despite the fact that At*Bna*DGAT2 contains both predicted transmembrane helices. Further investigation is thus necessary to fully understand the acyl-donor specificity and its impact on the catalytic activity.

The acyl-donor specificity may, of course, be the result of amino acids residing within the identified region, as well as amino acids residing elsewhere. The putative acyl acceptor pocket motif within the *Arabidopsis* DGAT2, for example, is constituted of nine amino acids distributed in a region of 72 amino acids according to a standard protein BLAST at NCBI (35). Further, the incorporation of different acyl-CoAs may depend on different amino acid residues. The approach implemented here is only possible because of the robust biochemical characterization of several DGAT2 enzymes that include the specificity toward 22:1-CoA. Biochemical determinations of at least five DGAT2 enzymes substrate specificities have so far identified these as good acceptors of 22:1-CoA (25, 26). It is, however, just as essential to have multiple DGAT2 enzymes characterized that do not accept 22:1-CoA to elucidate such a motif.

Elucidation of similar motifs governing acceptance for other unusual fatty acids, such as hydroxy fatty acids, is probably possible through targeted identification and biochemical characterization of DGAT2s from plant species with seed oils rich in such fatty acids. The DGAT2 of castor bean, for example, was characterized and identified already in 2006 and is efficient in incorporating ricinoleoyl containing substrates into TAG (11). There are several plant species within the genus *Lesquerella* known to produce only modest amounts of ricinoleic acid in their seed oils, but several of them produce seed oils rich in elongation products of ricinoleic acid. Characterization of *Lesquerella*-derived DGAT2 and analyses of their amino acid sequences compared with the castor bean DGAT may very well prove successful in the identification of yet another motif that catalyzes the incorporation of an unusual FA. As concluded in this study, implementation of substrate specificity modification is capable of affecting not only unusual FAs but also common FAs.

The strategy implanted here may elucidate yet another unresolved question regarding DGAT2, that of the acyl acceptor specificity. The putative acyl acceptor binding pocket situated in the approximate middle of the DGAT2 amino acid sequences may very well be the region where the determination of acyl acceptor specificity resides. There may, however, be unpredicted interactions between the two specificities affecting each other even though they may reside in different regions of the amino acid chain. In time, with the help of genetic engineering by, for example, CRISPR/Cas9, it may become possible to alter the substrate specificity of native DGAT2 enzymes. Such an approach would, in turn, open up a wide range of possibilities for efficient incorporation of novel FAs in existing oil crops. A chimeric enzyme with both modified acyl donor and acyl acceptor specificities would be a potent tool, exerting a specific selection for all three *sn* positions in TAG. A chimeric DGAT2 enzyme that efficiently incorporates a desired FA on *sn-*3 of specific desired DAG species may prove useful in the field of designed oil qualities.

## Materials and methods

### Constructs

Integrated DNA Technologies, Inc. provided full-length synthetic DNA; codon optimized for expression in yeast for *Bna*A.DGAT2.b, *Bna*A.DGAT2.d, AtDGAT2, and a full-length chimeric *Arabidopsis B. napus* At*Bna*DGAT2 sequence (sequences available in Refs. 26 and 36 and Fig. S2). *att*B sites flank the full-length DNA fragments that enable Gateway cloning. Integrated DNA Technologies, Inc. also provided chimeric gene fragments corresponding to the first 100 amino acids in *Bna*A.DGAT2 with the gateway cloning site *att*B1 added to the 5´ end (see Fig. S2 for all coding nucleotide sequences). The chimeric constructs were cloned by fusion PCR, during which the different fragments were fused with designated primers (see Fig. S3 for PCR primer sequences). Fusion PCR products with expected fragment size as estimated on a 1.2% agarose gel were excised and used for further cloning.

### Cloning and transformation

We used the Gateway cloning system (Invitrogen) to recombine full-length DGAT2 genes into entry vector pDONR221 and the yeast destination vector, pYES2-DEST52, according to the manufacturer's guidelines. All full-length genes were sequenced, once transformed into *Escherichia coli* TOP 10. Further, we transformed The GAL1::DGAT2 constructs contained within a destination vector into yeast strain H1246 (MATα *are1-*Δ::*HIS3 are2-*Δ::*LEU2 dga1-*Δ::*KanMX4 Iro-*Δ::*TRP1 ADE2 ura3*), otherwise incapable of TAG synthesis (37).

### Microsomal preparations

Expression of the DGAT2 genes was induced in liquid yeast cultures through substitution of glucose with galactose. The cultures were further incubated for ∼24 h before harvest. Microsomes were prepared through homogenization and centrifugation and stored at −80 °C until biochemical assays were conducted. Yeast cultures and microsomal preparations were carried out as described in Ref. 38.

### Chemicals

CoA and *Rhizomucor miehei* TAG lipase were acquired from Sigma–Aldrich; free FAs, 6:0, 18:1, 18:2, 18:3, 20:1, and 22:1, were procured from Larodan fine chemicals; and radiolabeled [^14^C]glycerol was provided from PerkinElmer. We produced [^14^C]di-6:0-DAG and unlabeled acyl-CoA as described in Ref. 25.

### Biochemical assays

Acyl-donor specificity was determined in microsomal preparations of recombinant yeast H1246, expressing either of the DGAT2 enzymes individually. Each assay consisted of 40 μg of microsomal protein, supplemented with 5 nmol [^14^C]di-6:0-DAG, 5 nmol acyl-CoA, and 1 mg BSA/ml final volume. The volume of each assay was adjusted with 50 mm HEPES buffer supplemented with 5 mm MgCl_2_, pH 7.2, to a final volume of 100 μl. The assays were incubated for 30 min at 30 °C with shaking at 1250 rpm. After termination of an assay with 500 μl of MeOH:CHCl_3_ (1:1, v/v) and 120 μl of 0.15 m acetic acid, the lipids were extracted into 250 μl chloroform according to (39). The total radioactivity was determined in an aliquot corresponding to one-fifth of the extracted lipids by liquid scintillation counting. We used either TLC or HPLC to separate the remaining four-fifths of the lipids, and TLC was used in assessments of the assays with the complete set of acyl-CoAs, whereas HPLC was used for the screening of 18:3-CoA and 22:1-CoA specificities. The relative contribution to the radioactive activity of the TLC separated radioactivity from each lipid class was determined by Instant Imager electronic autoradiograph, whereas the HPLC separated was detected by Raytest Ramona radioactivity detector. The amount of *de novo* formed TAG was determined by calculating the relative contribution of radioactive TAG and the total amount of radioactivity in the chloroform extract. For further details of TLC and HPLC separation, see Ref. 25.

### Alignments and transmembrane helix prediction

The alignment with two sequences was carried out with the Needleman–Wunsch algorithm and those with more than two sequences were carried out with the Clustal Omega algorithm (40, 41). Prediction of transmembrane helices was carried out with TMHMM (31).

## Data availability

The nucleotide sequences of the chimeras, primers, and nucleotide alignments are available in the supporting information. The original accessions used in the alignments are available at NCBI. Original sequences of WT parental sequences are available in Ref. 26.

## Supplementary Material

Supporting Information
