# Supplementary material for: A predicted transmembrane region in plant diacylglycerol acyltransferase 2 regulates specificity toward very-long-chain acyl-CoAs
Source: J Biol Chem. 2020 Sep 1;295(45):15398–406. doi: 10.1074/jbc.RA120.013755 (PMC7650248; doi:10.1074/jbc.RA120.013755)
Supplement: Supporting Information [file supp_295_45_15398__index.html]

A predicted transmembrane region in plant diacylglycerol acyltransferase 2 regulates specificity towards very long chain acyl-CoAs. — A delimited region in DGAT2 affects acyl donor specificity — A predicted transmembrane region in plant diacylglycerol acyltransferase 2 regulates specificity toward very-long-chain acyl-CoAs — A delimited region in DGAT2 affects acyl-donor specificity — Supporting Information 

# A predicted transmembrane region in plant diacylglycerol acyltransferase 2 regulates specificity toward very-long-chain acyl-CoAs

## Supporting Information

- Supplemental figure 1 - Alignment of DGAT2 sequences
- Supplemental figure 2 - Nucleotide sequences of the chimeric enzymes.
- Supplemental figure 3 - PCR primer sequences and primer combinations.
