# Supplementary material for: A predicted transmembrane region in plant diacylglycerol acyltransferase 2 regulates specificity toward very-long-chain acyl-CoAs
Source: J Biol Chem. 2020 Sep 1;295(45):15398–406. doi: 10.1074/jbc.RA120.013755 (PMC7650248; doi:10.1074/jbc.RA120.013755)
Supplement: Supporting Information [file supp_RA120.013755_159970_3_supp_585925_qfkdp3.pdf]

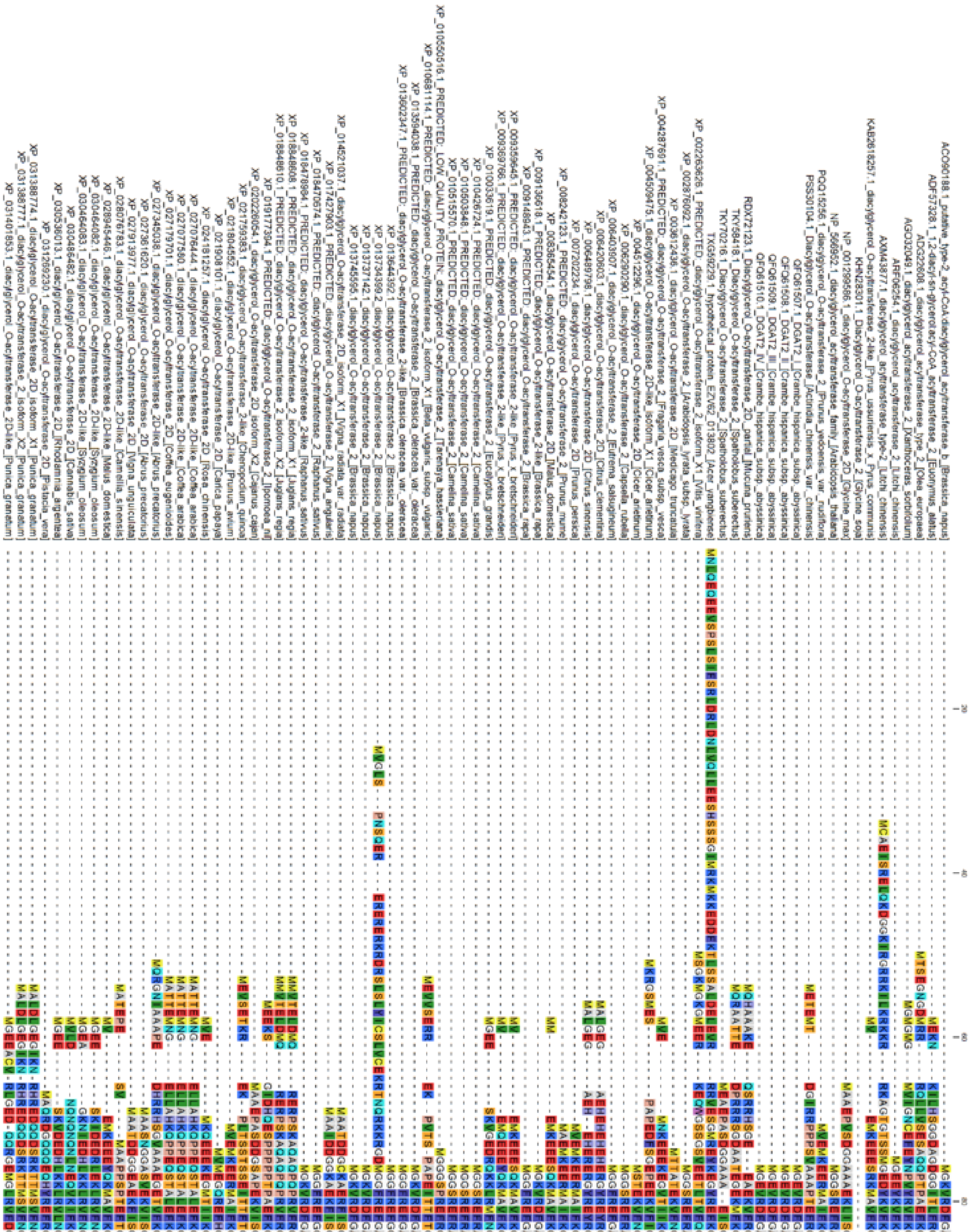

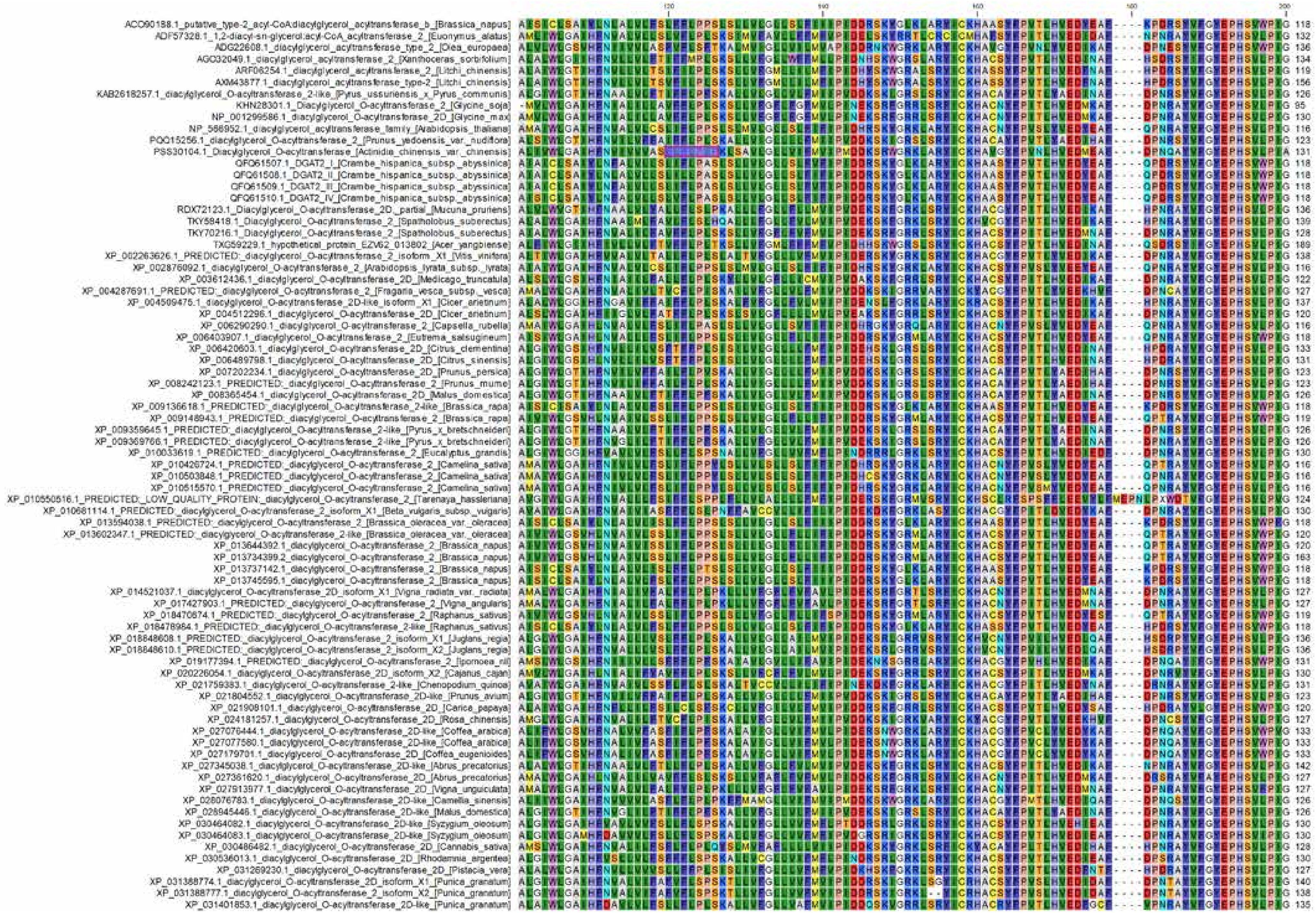

120
140
160
180
200
ACO90188.1_putative_type-2_acyl-CoA:diacylglycerol_acyltransferase_b_[Brassica_napus]
ADF57328.1_1,2-diacyl-sn-glycerol:acyl-CoA_acyltransferase_2_[Euonymus_alatus]
ADG22608.1_diacylglycerol_acyltransferase_type_2_[Olea_europaea]
AGO32049.1_diacylglycerol_acyltransferase_2_[Xanthoceras_sorbifolium]
ARF06254.1_diacylglycerol_acyltransferase_2_[Litchi_chinensis]
AXM43877.1_diacylglycerol_acyltransferase_type-2_[Litchi_chinensis]
KAB2618257.1_diacylglycerol_O-acyltransferase_2-like_[Pyrus_ussuriensis_x_Pyrus_communis]
KHN28301.1_Diacylglycerol_O-acyltransferase_2_[Glycine_soja]
NP_001299586.1_diacylglycerol_O-acyltransferase_2D_[Glycine_max]
NP_566952.1_diacylglycerol_acyltransferase_family_[Arabidopsis_thaliana]
PQQ15256.1_diacylglycerol_O-acyltransferase_2_[Prunus_yedoensis_var._nudiflora]
PSS30104.1_Diacylglycerol_O-acyltransferase_[Actinidia_chinensis_var._chinensis]
QFQ61507.1_DGAT2_I_[Crambe_hispanica_subsp._abyssinica]
QFQ61508.1_DGAT2_II_[Crambe_hispanica_subsp._abyssinica]
QFQ61509.1_DGAT2_III_[Crambe_hispanica_subsp._abyssinica]
QFQ61510.1_DGAT2_IV_[Crambe_hispanica_subsp._abyssinica]
RDX72123.1_Diacylglycerol_O-acyltransferase_2D_partial_[Mucuna_pruriens]
TKY58418.1_Diacylglycerol_O-acyltransferase_2_[Spatholobus_suberectus]
TKY70216.1_Diacylglycerol_O-acyltransferase_2_[Spatholobus_suberectus]
TXG59229.1_hypothetical_protein_EZV62_013802_[Acer_yangbiense]
XP_002263626.1_PREDICTED:_diacylglycerol_O-acyltransferase_2_isoform_X1_[Vitis_vinifera]
XP_002876092.1_diacylglycerol_O-acyltransferase_2_[Arabidopsis_lyrata_subsp._lyrata]
XP_003612436.1_diacylglycerol_O-acyltransferase_2D_[Medicago_truncatula]
XP_004287691.1_PREDICTED:_diacylglycerol_O-acyltransferase_2_[Fragaria_vesca_subsp._vesca]
XP_004509475.1_diacylglycerol_O-acyltransferase_2D-like_isoform_X1_[Cicer_arietinum]
XP_004512296.1_diacylglycerol_O-acyltransferase_2D_[Cicer_arietinum]
XP_006290290.1_diacylglycerol_O-acyltransferase_2_[Capsella_rubella]
XP_006403907.1_diacylglycerol_O-acyltransferase_2_[Eutrema_salsugineum]
XP_006420603.1_diacylglycerol_O-acyltransferase_2D_[Citrus_clementina]
XP_006489798.1_diacylglycerol_O-acyltransferase_2D_[Citrus_sinensis]
XP_007202234.1_diacylglycerol_O-acyltransferase_2D_[Prunus_persica]
XP_008242123.1_PREDICTED:_diacylglycerol_O-acyltransferase_2_[Prunus_mume]
XP_008365454.1_diacylglycerol_O-acyltransferase_2D_[Malus_domestica]
XP_009136618.1_PREDICTED:_diacylglycerol_O-acyltransferase_2-like_[Brassica_rapa]
XP_009148943.1_PREDICTED:_diacylglycerol_O-acyltransferase_2_[Brassica_rapa]
XP_009359645.1_PREDICTED:_diacylglycerol_O-acyltransferase_2-like_[Pyrus_x_bretschneideri]
XP_009369766.1_PREDICTED:_diacylglycerol_O-acyltransferase_2-like_[Pyrus_x_bretschneideri]
XP_010033619.1_PREDICTED:_diacylglycerol_O-acyltransferase_2_[Eucalyptus_grandis]
XP_010426724.1_PREDICTED:_diacylglycerol_O-acyltransferase_2_[Camelina_sativa]
XP_010503848.1_PREDICTED:_diacylglycerol_O-acyltransferase_2_[Camelina_sativa]
XP_010515570.1_PREDICTED:_diacylglycerol_O-acyltransferase_2_[Camelina_sativa]
XP_010550516.1_PREDICTED:_LOW_QUALITY_PROTEIN:_diacylglycerol_O-acyltransferase_2_[Tarenaya_hassleriana]
XP_010681114.1_PREDICTED:_diacylglycerol_O-acyltransferase_2_isoform_X1_[Beta_vulgaris_subsp._vulgaris]
XP_013594038.1_PREDICTED:_diacylglycerol_O-acyltransferase_2_[Brassica_oleracea_var._oleracea]
XP_013602347.1_PREDICTED:_diacylglycerol_O-acyltransferase_2-like_[Brassica_oleracea_var._oleracea]
XP_013644392.1_diacylglycerol_O-acyltransferase_2_[Brassica_napus]
XP_013734399.2_diacylglycerol_O-acyltransferase_2_[Brassica_napus]
XP_013737142.1_diacylglycerol_O-acyltransferase_2_[Brassica_napus]
XP_013745595.1_diacylglycerol_O-acyltransferase_2_[Brassica_napus]
XP_014521037.1_diacylglycerol_O-acyltransferase_2D_isoform_X1_[Vigna_radiata_var._radiata]
XP_017427903.1_PREDICTED:_diacylglycerol_O-acyltransferase_2_[Vigna_angularis]
XP_018470674.1_PREDICTED:_diacylglycerol_O-acyltransferase_2_[Raphanus_sativus]
XP_018478984.1_PREDICTED:_diacylglycerol_O-acyltransferase_2-like_[Raphanus_sativus]
XP_018848608.1_PREDICTED:_diacylglycerol_O-acyltransferase_2_isoform_X1_[Juglans_regia]
XP_018848610.1_PREDICTED:_diacylglycerol_O-acyltransferase_2_isoform_X2_[Juglans_regia]
XP_019177394.1_PREDICTED:_diacylglycerol_O-acyltransferase_2_[Ipomoea_nil]
XP_020226054.1_diacylglycerol_O-acyltransferase_2D_isoform_X2_[Cajanus_cajan]
XP_021759383.1_diacylglycerol_O-acyltransferase_2-like_[Chenopodium_quinoa]
XP_021804552.1_diacylglycerol_O-acyltransferase_2D-like_[Prunus_avium]
XP_021909101.1_diacylglycerol_O-acyltransferase_2D_[Carica_papaya]
XP_024181257.1_diacylglycerol_O-acyltransferase_2D_[Rosa_chinensis]
XP_027076444.1_diacylglycerol_O-acyltransferase_2D-like_[Coffea_arabica]
XP_027077580.1_diacylglycerol_O-acyltransferase_2D-like_[Coffea_arabica]
XP_027179701.1_diacylglycerol_O-acyltransferase_2D_[Coffea_eugenioides]
XP_027345038.1_diacylglycerol_O-acyltransferase_2D-like_[Abrus_precatorius]
XP_027361620.1_diacylglycerol_O-acyltransferase_2D_[Abrus_precatorius]
XP_027913977.1_diacylglycerol_O-acyltransferase_2D_[Vigna_unguiculata]
XP_028076783.1_diacylglycerol_O-acyltransferase_2D-like_[Camellia_sinensis]
XP_028945446.1_diacylglycerol_O-acyltransferase_2D-like_[Malus_domestica]
XP_030464082.1_diacylglycerol_O-acyltransferase_2D-like_[Syzygium_oleosum]
XP_030464083.1_diacylglycerol_O-acyltransferase_2D-like_[Syzygium_oleosum]
XP_030486462.1_diacylglycerol_O-acyltransferase_2D_[Cannabis_sativa]
XP_030536013.1_diacylglycerol_O-acyltransferase_2D_[Rhodamnia_argentea]
XP_031269230.1_diacylglycerol_O-acyltransferase_2D_[Pistacia_vera]
XP_031388774.1_diacylglycerol_O-acyltransferase_2D_isoform_X1_[Punica_granatum]
XP_031388777.1_diacylglycerol_O-acyltransferase_2_isoform_X2_[Punica_granatum]
XP_031401853.1_diacylglycerol_O-acyltransferase_2D-like_[Punica_granatum]

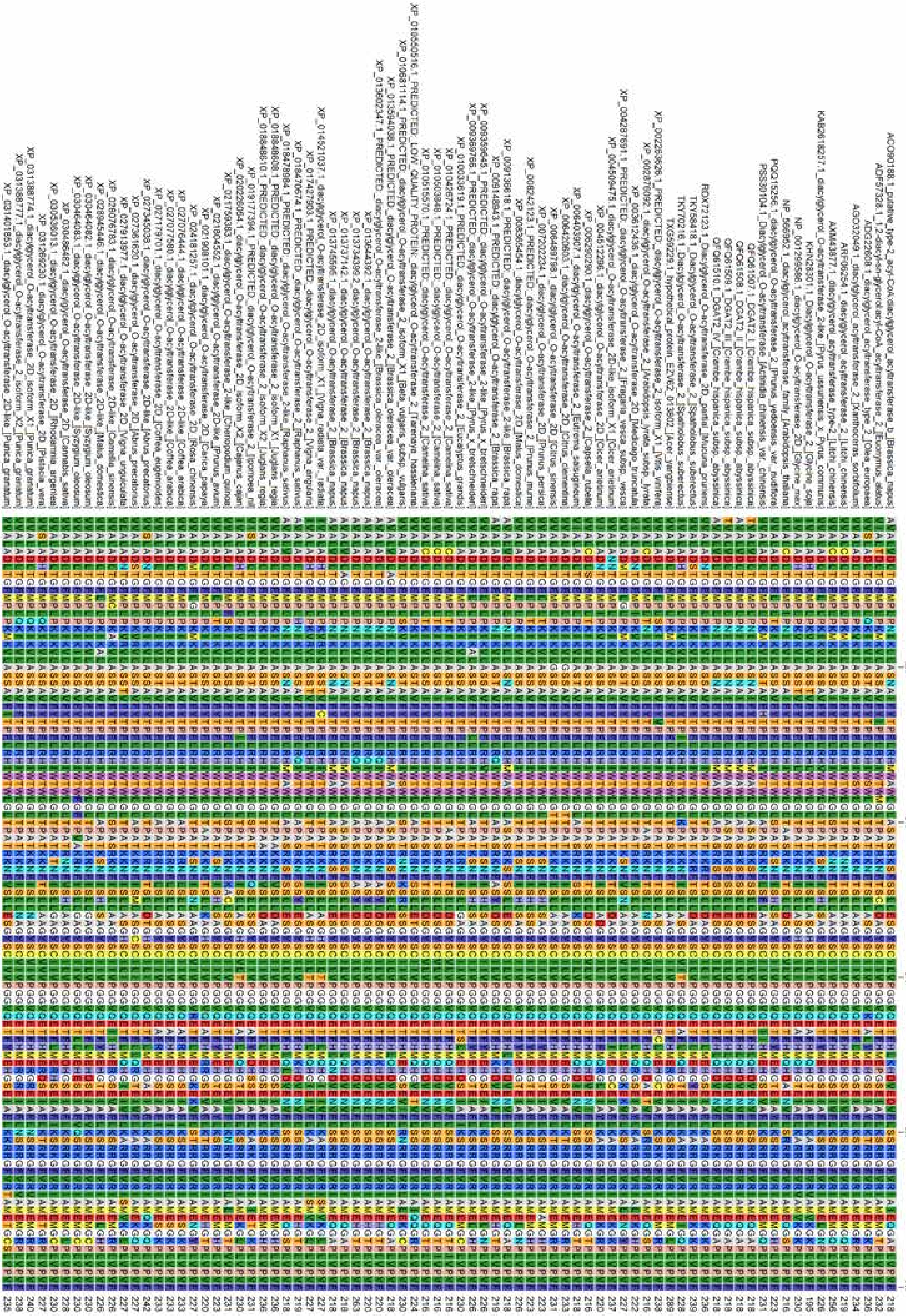

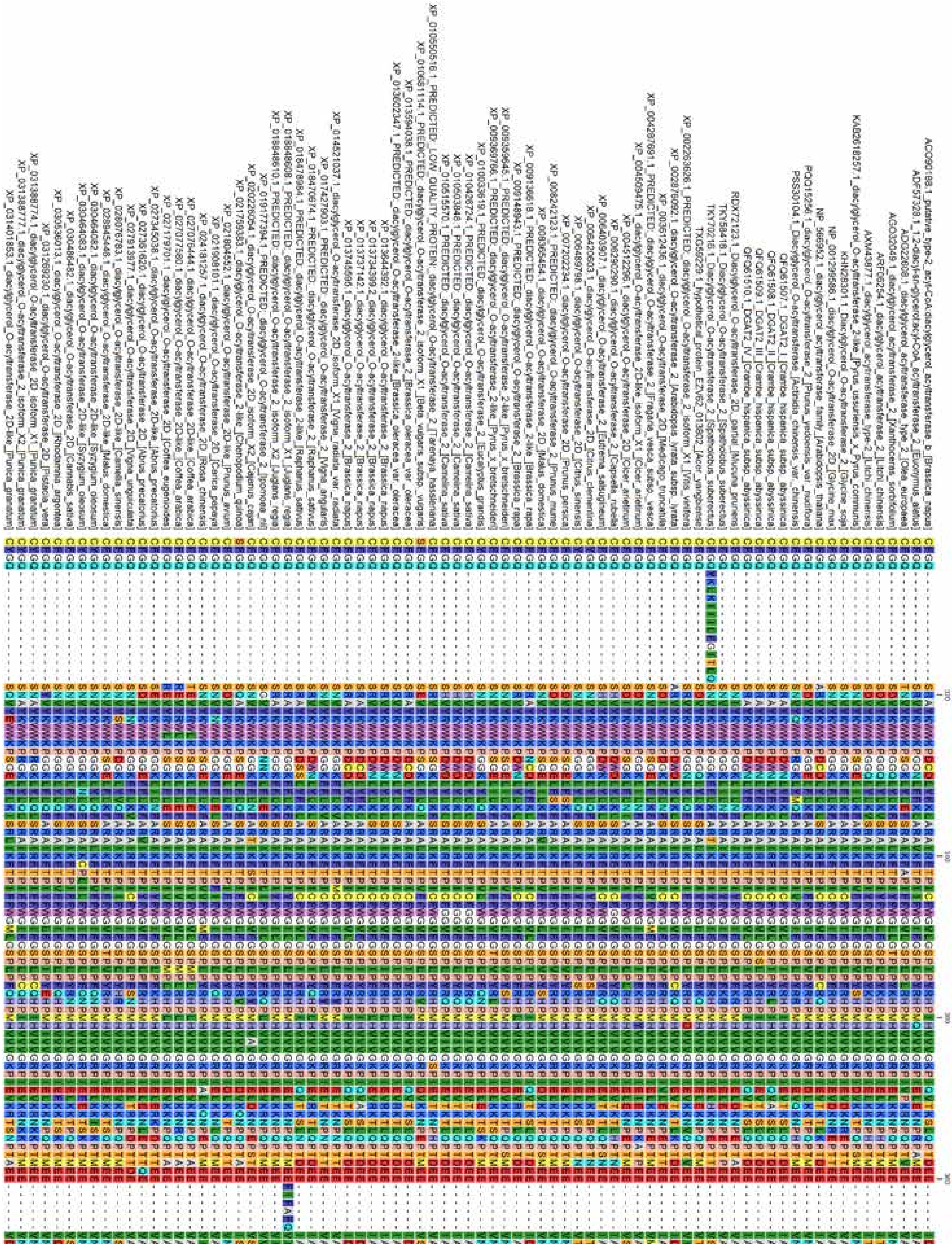

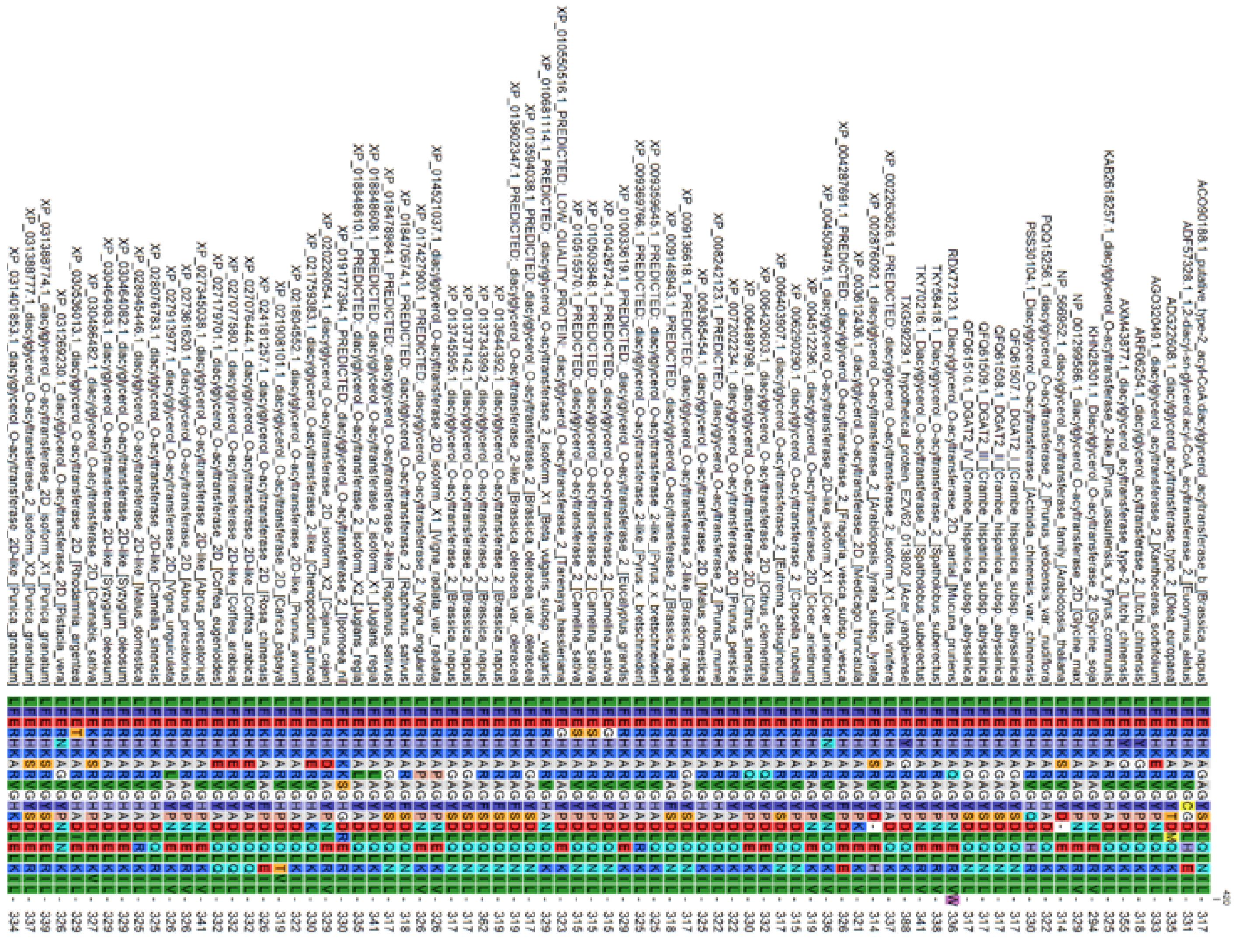

Supplemental 1 the alignment of 77 plant DGAT2 amino acid sequences produced by the ClustalW algorithm.
