# Supplementary material for: A predicted transmembrane region in plant diacylglycerol acyltransferase 2 regulates specificity toward very-long-chain acyl-CoAs
Source: J Biol Chem. 2020 Sep 1;295(45):15398–406. doi: 10.1074/jbc.RA120.013755 (PMC7650248; doi:10.1074/jbc.RA120.013755)
Supplement: Supporting Information [file supp_RA120.013755_159970_3_supp_585924_qfkdp3.pdf]

>bbb**d**:B:B

ATGGGTGGCTTTAGAGAGTTTGGCGGAGATGAGCACTCTTCATCATCTTCCAACCTATTC

CATTCTGTGACGGCTATCGTTATCTGGCTGGGGTCCGTGCATCTTAACGTTGCAATAGTC

ATATCAAGTCTGATCTTCTTGCCACCGAGCCTGTCTTTGTTACTGTTAGGACTTCTGTTT

CTTCTAATTTTCATTCCTATCGACGACAGATCTAAGTATGGCCTGAAATTGGCTAGATAC

ATCTGTAAGCACGCCGCGTCATACTTCCCCGTCACATTGCATGTAGAAGACTACGAGGCG

TTCCAACCCACCCGTGCGTACGTTTTTGGGTACGAACCCCACTCAGTGTGGCCGATAGGA

GCTGTTGCTTTGGCCGATCTTACGGGATTTATGCCTTTACCTAACATAAAAGTCCTAGCT

TCTACTGCCGTATTCTATACGCCGTTTCTGAGGCAAATATGGACATGGTTAGGATTGGCC

CCAGCTAGTAGAAAGAACTTTGCTAGCTACCTAGATTCTGGATATAGCTGCATACTTGTT

CCTGGGGGCGTCCAAGAGACGTTCCACATGAAACACGATGTTGAAAATTTATTTCTTAGC

TCACGTCGTGGATTTGTCAGAATCGCTATGGAACACGGGACGCCATTAGTCCCTGTATTT

TGCTTTGGACAAAGTAGGGTATATAAATGGTGGAAACCGGACTGGAACCTGTATTTGAAA

CTAAGCAGGGCAATAAAGTTTACCCCGATATGCTTTTGGGGCGTTTTTGGAAGTCCCATC

CCCTTCAGGCATCCACTGCACGTTGTTGTCGGCAAACCTATAGAGGTGCGTAAGACCTTA

CAGCCAACTGACGAAGAAATAGCAAAGGTGCATGGTCAGTTTGTCGAGGCATTAAAAGAC

CTTTTCGAAAGGCATAAAGCGAGAGCGGGTTTTTCCGATTTACAACTAAACATCCTTTGA

>bbdb:B:B

ATGGGTGGCTTTAGAGAGTTTGGCGGAGATGAGCACTCTTCATCATCTTCCAACCTATTC

CATTCTGTGACGGCTATCGTTATCTGGCTGGGGTCCGTGCATCTTAACGTTGCAATAGTC

ATATCAAGTCTGTTCTTCCTGCCACCGTCCTTATCATTGCTAGTACTGGGGTTGCTATCC

TTGTTTATAATAATCCCCATTGATGACAGAAGCAAATATGGTAGAATGTTAGCGCGTTAT

ATCTGCAAGCATGCGTGCTCATACTTTCCTGTAACCTTACACGTAGAAGATTATGAGGCC

TTCCAACCCACCCGTGCGTACGTTTTTGGGTACGAACCCCACTCAGTGTGGCCGATAGGA

GCTGTTGCTTTGGCCGATCTTACGGGATTTATGCCTTTACCTAACATAAAAGTCCTAGCT

TCTACTGCCGTATTCTATACGCCGTTTCTGAGGCAAATATGGACATGGTTAGGATTGGCC

CCAGCTAGTAGAAAGAACTTTGCTAGCTACCTAGATTCTGGATATAGCTGCATACTTGTT

CCTGGGGGCGTCCAAGAGACGTTCCACATGAAACACGATGTTGAAAATTTATTTCTTAGC

TCACGTCGTGGATTTGTCAGAATCGCTATGGAACACGGGACGCCATTAGTCCCTGTATTT

TGCTTTGGACAAAGTAGGGTATATAAATGGTGGAAACCGGACTGGAACCTGTATTTGAAA

CTAAGCAGGGCAATAAAGTTTACCCCGATATGCTTTTGGGGCGTTTTTGGAAGTCCCATC

CCCTTCAGGCATCCACTGCACGTTGTTGTCGGCAAACCTATAGAGGTGCGTAAGACCTTA

CAGCCAACTGACGAAGAAATAGCAAAGGTGCATGGTCAGTTTGTCGAGGCATTAAAAGAC

CTTTTCGAAAGGCATAAAGCGAGAGCGGGTTTTTCCGATTTACAACTAAACATCCTTTGA

>b**d**bb:B:B

ATGGGTGGCTTTAGAGAGTTTGGCGGAGATGAGCACTCTTCATCATCTTCCAACATTTTG

CATGCTGTGACGGCAATCTCAATCTGTCTGTCAGCAATTTATCTAAATCTAGCACTAGTA

TTGTTTTCTCTTATCTTCTTGCCACCGAGCCTGTCTTTGTTACTGTTAGGACTTCTGTTT

CTTCTAATTTTCATTCCTATCGACGACAGAAGCAAATATGGTAGAATGTTAGCGCGTTAT

ATCTGCAAGCATGCGTGCTCATACTTTCCTGTAACCTTACACGTAGAAGATTATGAGGCC

TTCCAACCCACCCGTGCGTACGTTTTTGGGTACGAACCCCACTCAGTGTGGCCGATAGGA

GCTGTTGCTTTGGCCGATCTTACGGGATTTATGCCTTTACCTAACATAAAAGTCCTAGCT

TCTACTGCCGTATTCTATACGCCGTTTCTGAGGCAAATATGGACATGGTTAGGATTGGCC

CCAGCTAGTAGAAAGAACTTTGCTAGCTACCTAGATTCTGGATATAGCTGCATACTTGTT

CCTGGGGGCGTCCAAGAGACGTTCCACATGAAACACGATGTTGAAAATTTATTTCTTAGC

TCACGTCGTGGATTTGTCAGAATCGCTATGGAACACGGGACGCCATTAGTCCCTGTATTT

TGCTTTGGACAAAGTAGGGTATATAAATGGTGGAAACCGGACTGGAACCTGTATTTGAAA

CTAAGCAGGGCAATAAAGTTTACCCCGATATGCTTTTGGGGCGTTTTTGGAAGTCCCATC

CCCTTCAGGCATCCACTGCACGTTGTTGTCGGCAAACCTATAGAGGTGCGTAAGACCTTA

CAGCCAACTGACGAAGAAATAGCAAAGGTGCATGGTCAGTTTGTCGAGGCATTAAAAGAC

CTTTTCGAAAGGCATAAAGCGAGAGCGGGTTTTTCCGATTTACAACTAAACATCCTTTGA

>b**dd**b:B:B

ATGGGTGGCTTTAGAGAGTTTGGCGGAGATGAGCACTCTTCATCATCTTCCAACATTTTG

CATGCTGTGACGGCAATCTCAATCTGTCTGTCAGCAATTTATCTAAATCTAGCACTAGTA

TTGTTTTCTCTTTTCTTCCTGCCACCGTCCTTATCATTGCTAGTACTGGGGTTGCTATCC

TTGTTTATAATAATCCCCATTGATGACAGAAGCAAATATGGTAGAATGTTAGCGCGTTAT

ATCTGCAAGCATGCGTGCTCATACTTTCCTGTAACCTTACACGTAGAAGATTATGAGGCC

TTCCAACCCACCCGTGCGTACGTTTTTGGGTACGAACCCCACTCAGTGTGGCCGATAGGA

GCTGTTGCTTTGGCCGATCTTACGGGATTTATGCCTTTACCTAACATAAAAGTCCTAGCT

TCTACTGCCGTATTCTATACGCCGTTTCTGAGGCAAATATGGACATGGTTAGGATTGGCC

CCAGCTAGTAGAAAGAACTTTGCTAGCTACCTAGATTCTGGATATAGCTGCATACTTGTT

CCTGGGGGCGTCCAAGAGACGTTCCACATGAAACACGATGTTGAAAATTTATTTCTTAGC

TCACGTCGTGGATTTGTCAGAATCGCTATGGAACACGGGACGCCATTAGTCCCTGTATTT

TGCTTTGGACAAAGTAGGGTATATAAATGGTGGAAACCGGACTGGAACCTGTATTTGAAA

CTAAGCAGGGCAATAAAGTTTACCCCGATATGCTTTTGGGGCGTTTTTGGAAGTCCCATC

CCCTTCAGGCATCCACTGCACGTTGTTGTCGGCAAACCTATAGAGGTGCGTAAGACCTTA

CAGCCAACTGACGAAGAAATAGCAAAGGTGCATGGTCAGTTTGTCGAGGCATTAAAAGAC

CTTTTCGAAAGGCATAAAGCGAGAGCGGGTTTTTCCGATTTACAACTAAACATCCTTTGA

>D:B:B

ATGGGGAAAGTCCGTGACTTTGGTGCTGAAGATCATATACCCTCTAACATTTTGCATGCT

GTGACGGCAATCTCAATCTGTCTGTCAGCAATTTATCTAAATCTAGCACTAGTATTGTTT

TCTCTTTTCTTCCTGCCACCGTCCTTATCATTGCTAGTACTGGGGTTGCTATCCTTGTTT

ATAATAATCCCCATTGATGACAGATCTAAGTATGGCCTGAAATTGGCTAGATACATCTGT

AAGCACGCCGCGTCATACTTCCCCGTCACATTGCATGTAGAAGACTACGAGGCGTTCCAA

CCCACCCGTGCGTACGTTTTTGGGTACGAACCCCACTCAGTGTGGCCGATAGGAGCTGTT

GCTTTGGCCGATCTTACGGGATTTATGCCTTTACCTAACATAAAAGTCCTAGCTTCTACT

GCCGTATTCTATACGCCGTTTCTGAGGCAAATATGGACATGGTTAGGATTGGCCCCAGCT

AGTAGAAAGAACTTTGCTAGCTACCTAGATTCTGGATATAGCTGCATACTTGTTCCTGGG

GGCGTCCAAGAGACGTTCCACATGAAACACGATGTTGAAAATTTATTTCTTAGCTCACGT

CGTGGATTTGTCAGAATCGCTATGGAACACGGGACGCCATTAGTCCCTGTATTTTGCTTT

GGACAAAGTAGGGTATATAAATGGTGGAAACCGGACTGGAACCTGTATTTGAAACTAAGC

AGGGCAATAAAGTTTACCCCGATATGCTTTTGGGGCGTTTTTGGAAGTCCCATCCCCTTC

AGGCATCCACTGCACGTTGTTGTCGGCAAACCTATAGAGGTGCGTAAGACCTTACAGCCA

ACTGACGAAGAAATAGCAAAGGTGCATGGTCAGTTTGTCGAGGCATTAAAAGACCTTTTC

GAAAGGCATAAAGCGAGAGCGGGTTTTTCCGATTTACAACTAAACATCCTTTGA

>**ddd**b:B:B

ATGGGGAAAGTCCGTGACTTTGGTGCTGAAGATCATATACCCTCTAACATTTTGCATGCT

GTGACGGCAATCTCAATCTGTCTGTCAGCAATTTATCTAAATCTAGCACTAGTATTGTTT

TCTCTTTTCTTCCTGCCACCGTCCTTATCATTGCTAGTACTGGGGTTGCTATCCTTGTTT

ATAATAATCCCCATTGATGACAGAAGCAAATATGGTAGAATGTTAGCGCGTTATATCTGC

AAGCATGCGTGCTCATACTTTCCTGTAACCTTACACGTAGAAGATTATGAGGCCTTCCAA

CCCACCCGTGCGTACGTTTTTGGGTACGAACCCCACTCAGTGTGGCCGATAGGAGCTGTT

GCTTTGGCCGATCTTACGGGATTTATGCCTTTACCTAACATAAAAGTCCTAGCTTCTACT

GCCGTATTCTATACGCCGTTTCTGAGGCAAATATGGACATGGTTAGGATTGGCCCCAGCT

AGTAGAAAGAACTTTGCTAGCTACCTAGATTCTGGATATAGCTGCATACTTGTTCCTGGG

GGCGTCCAAGAGACGTTCCACATGAAACACGATGTTGAAAATTTATTTCTTAGCTCACGT

CGTGGATTTGTCAGAATCGCTATGGAACACGGGACGCCATTAGTCCCTGTATTTTGCTTT

GGACAAAGTAGGGTATATAAATGGTGGAAACCGGACTGGAACCTGTATTTGAAACTAAGC

AGGGCAATAAAGTTTACCCCGATATGCTTTTGGGGCGTTTTTGGAAGTCCCATCCCCTTC

AGGCATCCACTGCACGTTGTTGTCGGCAAACCTATAGAGGTGCGTAAGACCTTACAGCCA

ACTGACGAAGAAATAGCAAAGGTGCATGGTCAGTTTGTCGAGGCATTAAAAGACCTTTTC

GAAAGGCATAAAGCGAGAGCGGGTTTTTCCGATTTACAACTAAACATCCTTTGA

>At*Bna*DGAT2

ATGGGTGGTTCTAGAGAGTTTAGGGCTGAAGAGCATATACCCTCTAACATTTTGCATGCT

GTGACGGCAATCTCAATCTGTCTGTCAGCAATTTATCTAAATCTAGCACTAGTATTGTTT

TCTCTTTTCTTCCTGCCACCGTCCTTATCATTGCTAGTACTGGGGTTGCTATCCTTGTTT

ATAATAATCCCCATTGATCATAGGAGCAAGTATGGCAGGAAATTAGCCAGATATATCTGC

AAACATGCATGTAATTACTTTCCAGTTTCCTTGTACGTGGAAGATTACGAAGCCTTTCAA

CCAAATAGAGCGTATGTGTTTGGATACGAACCCCATAGTGTTTTGCCAATTGGTGTTGTA

GCCTTGTGTGACTTGACAGGCTTCATGCCTATTCCCAACATTAAGGTGCTTGCTTCATCT

GCTATATTTTACACTCCATTTCTACGTCACATTTGGACTTGGCTAGGTTTGACAGCTGCT

AGTCGTAAGAACTTCACATCTCTGTTGGATTCCGGTTATTCTTGTGTTCTAGTTCCTGGT

GGTGTCCAAGAAACGTTTCACATGCAGCATGATGCTGAAAACGTTTTCCTATCGAGAAGA

AGAGGCTTTGTACGTATAGCAATGGAACAAGGAAGTCCTTTAGTCCCTGTCTTTTGTTTC

GGTCAAGCTAGAGTCTATAAATGGTGGAAACCTGATTGCGACTTGTACTTAAAGTTGTCT

AGGGCAATTAGATTTACTCCTATTTGCTTTTGGGGAGTATTTGGTTCACCGCTTCCATGT

AGACAACCAATGCATGTTGTTGTGGGAAAACCGATTGAAGTAACCAAGACCTTAAAACCC

ACTGATGAAGAGATAGCGAAATTTCATGGGCAATATGTAGAAGCCTTGAGAGACTTATTC

GAGAGACACAAATCCAGAGTGGGCTATGATCTTGAACTGAAGATACTATAG

>BnaDGAT2 mutated

ATGGGGAAAGTCCGTGACTTTGGTGCTGAAGATCATATACCCTCTAACATTTTGCATGCTGTGACGGCAATCTC
AATCTGTCTGTCAGCAATTTATCTAAATCTAGCACTAGTATTGTTTTCTCTTTTCTTCCTGCCACCGTCCTTATC
ATTGCTAGTACTGGGGTTGCTATCCTTGTTTATAATAATCCCCATTGATGACAGATCTAAGTATGGCCTGAAAT
TGGCTAGATACATCTGTAAGCACGCCGCGTCATACTTCCCCGTCACATTGCATGTAGAAGACTACGAGGCGTT
CAAGCCCGACAGGAGTTACGTCTTCGGTTACGAGCCGCACTCCGTGTGGCCGATCGGGGCTGTAGCCCTGGTA
GACCTAACTGGTTTCATGCCTTTACCTAACATTAAGTTATTGGCTTCTAACGCGATATTCTACACACCATTCCT
ACGTCACATGTGGGCCTGGTTAGGCTTGGCCTCAGCGTCCCGTAAAAGTTTTTCCAGCCTATTAGAGAGTGGCT
ACTCATGCATTCTAGTGCCTGGAGGAGTACAGGAAACATTTCACCTTCAGAATGATGTCGAGAATGTCTTCTT
GAGCTCACGTAGAGGATTTGTTCGTATAGCAATGGAACAAGGTGCTCCTCTGGTCCCGGTTTTTTGTTTTGGGC
AAAGTAGAGCGTATAAGTGGTGGAAGCCGGATTGCGATTTGTATTTTAAGTTGGCGAGAGCAATAAGGTTCAC
GCCAATCTGCTTCTGGGGGGTATTAGGTTCTCCCATCCCTTACAGGCACCCTATACATGTAGTAGTGGGGAAA

CCCATACAAGTCACGAAAAGTTTGCAACCGACTGACGAGGAAATTGACGAGTTGCATGGGCAATTTGTCGAG
GCATTAAAGGACTTATTTGAAAGACATAAGGCTGGAGCGGGTTATAGCGATCTTCAGTTAAATATACTGTAA

Supplemental 2 nucleotide sequences of chimeric enzymes
