# Supplementary material for: A predicted transmembrane region in plant diacylglycerol acyltransferase 2 regulates specificity toward very-long-chain acyl-CoAs
Source: J Biol Chem. 2020 Sep 1;295(45):15398–406. doi: 10.1074/jbc.RA120.013755 (PMC7650248; doi:10.1074/jbc.RA120.013755)
Supplement: Supporting Information [file supp_RA120.013755_159970_3_supp_585923_qfkdp3.pdf]

primer name	nucleotide sequence
*BnaA.DGAT2.**d** F*	GGGGACAAGTTTGTACAAAAAAGCAGGCTATGGGGAAAGTCCGTGA
BnaA.DGAT2.b F	GGGGACAAGTTTGTACAAAAAAGCAGGCTATGGGTGGCTTTAGAGAGTTT
*BnaA.DGAT2.**d** R*	GGGGACCACTTTGTACAAGAAAGCTGGGTTTACAGTATATTTAACTGAAGATCGCT
BnaA.DGAT2.b R	GGGGACCACTTTGTACAAGAAAGCTGGGTTCAAAGGATGTTTAGTTGTAAATCG
*BnaA.DGAT2 **d**/B F*	CATGTAGAAGACTACGAGGCGTTCCAACCCACCCGT
*BnaA.DGAT2 **d**/B R*	ACGGGTGGGTTGGAACGCCTCGTAGTCTTCTACATG
BnaA.DGAT2 b/B F	TACACGTAGAAGATTATGAGGCCTTCCAACCCACCCGT
BnaA.DGAT2 b/B R	ACGGGTGGGTTGGAAGGCCTCATAATCTTCTACGTGTA

	**D**:B:B		bbbd:B:B		**ddd**b:B:B
**template 1**	BnaA.DGAT2.d	**template 1**	Gblock	**template 1**	Gblock
	*BnaA.DGAT2.**d** F*		*BnaA.DGAT2.b F*		*BnaA.DGAT2.**d** F*
	*BnaA.DGAT2 **d**/b R*		*BnaA.DGAT2 **d**/b F*		*BnaA.DGAT2 b/B R*
**template 2**	BnaA.DGAT2.b	**template 2**	BnaA.DGAT2.b	**template 2**	BnaA.DGAT2.b
	*BnaA.DGAT2 **d**/B F*		*BnaA.DGAT2 **d**/B F*		*BnaA.DGAT2 b/B F*
	BnaA.DGAT2.b R		*BnaA.DGAT2.b R*		*BnaA.DGAT2.b R*
	**Fusion**		**Fusion**		**Fusion**
	*BnaA.DGAT2.**d** F*		*BnaA.DGAT2.b F*		*BnaA.DGAT2.**d** F*
	BnaA.DGAT2.b R		*BnaA.DGAT2.b R*		*BnaA.DGAT2.b R*

	b**dd**b:B:B		b**d**bb:B:B		bb**d**b:B:B
**template 1**	Gblock	**template 1**	Gblock	**template 1**	Gblock
	BnaA.DGAT2.b F		*BnaA.DGAT2.b F*		*BnaA.DGAT2.b F*
	BnaA.DGAT2 b/B R		*BnaA.DGAT2 b/B R*		*BnaA.DGAT2 b/B R*
**template 2**	BnaA.DGAT2.b	**template 2**	BnaA.DGAT2.b	**template 2**	BnaA.DGAT2.b
	BnaA.DGAT2 b/B F		BnaA.DGAT2 b/B F		BnaA.DGAT2 b/B F
	BnaA.DGAT2.b R		BnaA.DGAT2.b R		BnaA.DGAT2.b R
	**Fusion**		**Fusion**		**Fusion**
	BnaA.DGAT2.b F		BnaA.DGAT2.b F		BnaA.DGAT2.b F
	BnaA.DGAT2.b R		BnaA.DGAT2.b R		BnaA.DGAT2.b R

Supplemental 3 primers and primer combinations
